# Race as a Contributor to Stromal Modulation of Tumor Progression

**DOI:** 10.3390/cancers13112656

**Published:** 2021-05-28

**Authors:** Mamatha Kakarla, Sathyavathi ChallaSivaKanaka, Simon W. Hayward, Omar E. Franco

**Affiliations:** Department of Surgery, NorthShore University HealthSystem, Research Institute, 1001 University Place, Evanston, IL 60201, USA; mkakarla@northshore.org (M.K.); SChalla@northshore.org (S.C.); SHayward@northshore.org (S.W.H.)

**Keywords:** tumor microenvironment, cancer racial disparity, African American, health disparity, stromal cells, immune suppression, treatment outcome, therapeutics, prostate cancer, breast cancer, mortality

## Abstract

**Simple Summary:**

When compared to European Americans (EA), the African American (AA) population is at a higher risk of developing various forms of cancers and is more vulnerable to cancer-related death. To overcome these disparities and develop personalized treatment strategies, it is important to understand the factors contributing to tumor progression and aggressiveness in AA patients. The tumor microenvironment (TME) contains various cellular and non-cellular components known to play an important role in tumor growth and progression. Recent studies indicate racial differences in gene expression within the TME. In this review, we focus on such differences in various cancers and discuss the relevance of this TME diversity in the acquisition of aggressive forms of disease and poorer response to therapy in AA patients. In general, AA patients appear to host a more immune suppressive TME, suggesting the potential utility of targeting this aspect of tumor biology.

**Abstract:**

Stromal cells play crucial roles in tumor development and are increasingly attractive targets for therapy. There are considerable racial disparities in the incidence and progression of many tumors, reflecting both environmental exposure and genetic differences existing between races. Tumorigenesis and tumor progression are linked to both the propensity to suffer an initiating event and the host response to such an event once it occurs, contributing to incidence and outcomes. In this review, we focused on racial disparities in the tumor microenvironment (TME) of different cancers as potential modulators of growth, metastasis, and response to treatment. Several studies suggest that the TME in AA has a distinct tumor biology and may facilitate both early onset and aggressive tumor growth while inhibiting anti-tumorigenic properties. The TME of AA patients often exhibits an immunosuppressive microenvironment with a substantial enrichment of immune inflammatory pathways and genes. As a result, AA patients can potentially benefit more from treatment strategies that modulate the immune system. Focusing on TME components for diagnostic and therapeutic purposes to address racial disparities is a promising area of investigation. Future basic and clinical research studies on personalized cancer diagnosis and treatment should acknowledge the significance of TME in racial disparities.

## 1. Introduction

**Figure 1:**
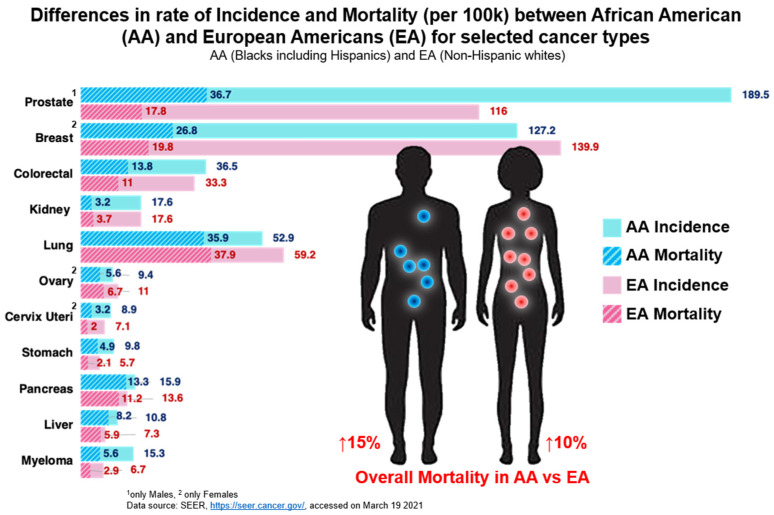
Differences in rate of incidence and mortality between African Americans and European Americans for selected cancer types.

In the USA, African Americans (AA) constitute only 12.5% of total US population but are disproportionately affected by cancer. The death rate due to cancer at all sites combined is 169.1 per 100,000 for AAs compared to 150.2 per 100,000 for European Americans (EAs) [1]. Despite the significant improvements in cancer diagnosis and treatment strategies in the past few decades, the mortality rate and development of aggressive forms of disease among the AA population remain higher compared to other races. These racial disparities are seen in various types of cancer (Figure 1). Overall, the mortality rate due to cancer in the AA male population is 15% higher than EA men and 10% higher in AA women compared to EA women [1].

To overcome these disparities and develop personalized treatment strategies, it is important to understand the factors contributing to tumor progression and metastasis in the AA population. Racial disparities in cancer development can be broadly influenced by non-biological and biological effects. Epidemiological studies illuminate the impact of exogenous "non-biological” factors on health disparities in cancer. For example, the incidence and mortality of various types of cancers are greatly influenced by factors including socio-economic conditions, behavioral/lifestyle practices, and environmental conditions. Recent data from the National Cancer Institute (NCI) show that all of these conditions contribute to racial disparities with minority groups (such as AA, Hispanics, American Indians and Alaska natives, Asians, and Pacific Islanders) being the most affected [1,2]. Several studies have shown that income [3,4,5,6] and access to healthcare [7] are significantly associated with cancer mortality rates among AA men and women. Pre-existing conditions such as hypertension, diabetes mellitus, obesity, chronic kidney disease, and cardiovascular diseases can complicate the treatment of several types of cancers [8,9,10]. These pre-existing conditions are more common in minorities and low-income populations, and they may also play a role in cancer health disparities [11]. Although non-biological factors contribute to racial disparities in the US, they do not completely explain the incidence and mortality in AA compared to EA [12,13,14]. After adjusting for the aforementioned socio-economic and cultural factors, several epidemiological and comparative studies have suggested the potential role of biological factors contributing to racial disparity in cancer [12,13].

Endogenous, “biological” factors include systemic genomic and genetic differences, miRNA alterations, epigenetic changes, and alterations to cellular signaling pathways, all which feed into the nature of the cancer cells and their tumor microenvironment (TME) that develops. It is undeniable that many of the exogenous factors could induce somatic, genetic, as well as epigenetic changes occurring within and around cancer cells to alter the biology of tumors and affect racial disparities in incidence and progression. The scientific literature is filled with genetic studies looking at the mutational burden of tumors or genome-wide association studies (GWAS) to explain racial disparities in cancer [15,16,17]. There is increasing evidence supporting the notion that the TME may contribute to racial disparities observed in the incidence and outcomes of different types of cancer reported in the AA compared to the EA population [18,19,20,21,22]. In this review, we address TME biology as a component of racial disparities research and provide a summary of recent studies highlighting the microenvironment as a modulator of tumor growth, metastasis, and response to treatment.

## 2. Tumor Microenvironment (TME)

The last decade has seen an explosion in basic research surrounding the TME and its components, exploring its role in cancer development and progression. The resultant discoveries have opened the door to the development of novel therapies targeting key components of the TME that could revolutionize cancer treatment. The TME actively participates during the development and progression of many types of cancer through the involvement of a range of its cellular components including cancer associated fibroblasts (CAF), endothelial cells, pericytes, immune-inflammatory cells, as well as non-cellular extracellular matrix (ECM) components such as collagen, fibronectin, and laminin [23,24,25]. The TME can positively or negatively regulate cancer cell invasion and metastasis by releasing cytokines, growth factors, and modifying cellular adhesion and behavior [23,26].

The TME is heterogeneous in nature and develops as a result of host interactions with cancer cells. Stromal–epithelial interactions facilitate cancer progression in a bi-directional manner either by direct cell contact or by paracrine/exocrine signaling [27,28]. As tumors progress in both grade and stage the TME evolves and is modified [29]. CAF are an abundant cell type in TME and are one of the key components that orchestrate tumorigenesis and metastasis by various mechanisms. CAF secrete growth factors and cytokines such as transforming growth factor- β (TGF- β), platelet derived growth factor (PDGF), hepatocyte growth factor (HGF), and vascular endothelial growth factor (VEGF) that modify the TME and enhance cancer cell growth [30,31,32,33,34,35,36]. Cancer cell motility can be influenced by the CAF expression of chemotactic chemokines such as stromal derived factor (SDF-1 or CXCL12), and C-C Motif Chemokine Ligand 2 (CCL2). Recent studies from our laboratory in prostate cancer (PCa) show that the secretion of chemokines by CAF enhance recruitment of inflammatory cells and promotes macrophage migration [37]. The activation of CXCL12 and its receptor CXC chemokine receptor 4 (CXCR4) signaling has been shown to promote cancer cell growth and metastasis in various types of cancers [38,39,40,41].

Cells within the TME communicate with each other via different mechanisms and these interactions play a key role in tumorigenesis, metastases, and drug resistance. For example, CAFs interact with other stromal cells such as pericytes and endothelial cells, promoting invasion and metastasis in various cancers. These interactions are mediated by secretion of chemokines and growth factors such as CXCL12, VEGF, fibroblast growth factor 2 (FGF-2), and PDGF [42]. These inter-cellular communications within TME as well as stromal cross talk with cancer cells remodel the ECM by mechanisms including cross-link, deposition or physical remodeling [43], providing a fertile soil for tumor progression.

Tumor-infiltrating immune cells are another major component of the TME and have gained significant attention in recent years for their important role in tumorigenesis and immunotherapy. Monocytes and the classical subsets of macrophages (M0, M1, and M2) comprise a significant portion of the leukocytes recruited in the vicinity of cancer cells. In the TME, tumor associated macrophages (TAM) are converted from a tumor suppressive M1 to an immunosuppressive and tumor promoting M2 phenotype. These M2 macrophages secrete a profile of factors that have been associated with growth and metastasis in several types of cancers [44,45]. In addition to macrophages, the presence CD8+ T cells and CD4+ T helper 1 cells (TH1) and their secreted cytokines such as interleukin-2 (IL-2), and interferon-γ (IFN γ) are often associated with good prognosis in various cancer types. On the other hand, high amounts of CD4+ T helper 2 cells (TH2) in tumors can promote metastases.

The TME reflects both the response of a given patient to a tumor and the nature of the tumor itself. As such, there are likely individual and racial variations reflecting both genetics and environmental influences that determine the nature of the specific microenvironmental responses seen in cancer [46,47]. Because the TME is a key player in tumor biology, in the following section, we summarize its role in various types of cancer in relation to racial disparities. In most cancers, the AA population has the highest mortality rates (Figure 1), therefore better understanding of racial differences in the TME could aid in the identification of novel diagnosis markers and tailored therapeutics to reduce the adverse outcomes of cancer in the AA population.

## 3. Race, TME and Tumor Site

Racial disparities have been observed in many different types of cancers. To better illustrate these differences, individual affected sites are classified based on organ systems and highlighting those with the most scientific literature available followed by understudied tumors.

### 3.1. Reproductive System

#### 3.1.1. Prostate

Prostate cancer is the most common non-skin cancer among men in the US and was diagnosed in 207,430 men in 2017 [2]. According to the most recent available data, 30,486 men died of PCa in the year 2017 in the US [1]. Although the overall incidence of PCa is declining with an estimated 191,930 cases in 2020, the mortality continues to increase with an estimated 33,330 deaths [1]. The rate of transformation of PCa from a latent to an aggressive invasive disease is significantly higher in AA compared to EA [48].

The prostate gland is rich in stromal elements that govern epithelial function in a benign state and contribute to the malignant transformation, growth, and invasion of cancer cells [49]. Studies suggest the presence of distinct gene expression profiles of immune cells, fibroblasts, and vascular components between the TME of AA men and EA men with PCa [18,22,50,51,52]. Work by Cher et al. in the late 1990s looking at primary PCa tumors found similar patterns of chromosomal alterations (more than 90% congruence) between AA and EA [53]. However, the gene expression profiles did differ significantly between the two racial groups, implicating genes involved in immune response and activation of pathways associated with metastasis [22,51]. Among these, the expression of phosphoserine phosphatase like (PSPHL) and Beta-crystallin B2 (CRYBB2) were significantly elevated in the tumor stroma of AA men compared to EA patients [22,51]. A higher expression of PSPHL and CRYBB2 in AA patients is also associated with cancer disparity in other malignancies including breast [54], colorectal [55], and endometrial [56], cancers. These gene products play an important role in tumor-stroma crosstalk during disease progression and in regulating immune response [57,58]. PSPH is known to promote tumor progression and is associated with poor prognosis in non-small cell lung cancer. However, the functional role of these molecules in prostate tissues and their association with PCa has not been studied. An intronic single nucleotide polymorphism (SNP rs9608380) on the CRYBB2 gene, a potentially functional variant, is associated with increased risk of PCa in AA men [59]. Interestingly, the overexpression of the CRYBB2 gene in triple negative breast cancer cells promoted tumor progression by increasing growth, invasiveness, IL6 production, immune cell chemo attraction, and the expression of metastasis-associated genes [57]. The functional consequences of PSPHL and CRYBB2 on PCa racial disparities remain to be evaluated.

More recently, the analysis of gene expression profiles from PCa tissues indicated prominent differences in tumor immunobiology between AA and EA men. For example, higher expressions of metastasis associated genes such as Autocrine Motility Factor Receptor (AMFR), CXCR4, C-C Motif Chemokine Receptor 7 (CCR7), and Matrix metallopeptidase 9 (MMP9) were found in the TME of AA patients compared to EA [22]. These gene products are known to be involved in the activation of pro-inflammatory pathways by mobilization and polarization of macrophages in the TME [60]. In another study, Kinseth et al., using laser capture microdissection of PCa specimens, identified 677 genes that were differentially expressed in the PCa stroma between AA and EA men with localized disease [51]. This list also includes genes associated with immune response and, in addition, a set of molecules that regulate cell adhesion, stress fiber formation, cytoskeletal remodeling, and epithelial-mesenchymal transition. It was noted that several genes involved in cell adhesion, and cytoskeletal remodeling pathways (NCK2, ROCK2, VCL, PARVA, ACTN, ARP2/3 and NID1, PPARD, TCF4, FN1, as well as several collagen and integrin genes) were upregulated in the tumor stroma of EA men compared to AA men [51]. These pathways are recognized to play an important role in cancer cell migration and metastases, and lower titers in AA men could enable epithelial-mesenchymal transition to facilitate the motility of cancer cells. Furthermore, reduced levels of genes involved in cell adhesion and stress fiber formation in the stroma of AA patients could possibly lead to a more aggressive form of PCa in this racial group [51].

CAF are a major component of tumor stromal cells and their involvement in prostate tumor development and progression is executed through stromal-epithelial cell interactions [30,61,62]. PCa cells exposed to conditioned media from prostate primary fibroblasts isolated from AA patients with localized PCa have shown increased in vitro proliferation and migration compared to those exposed from EA prostate fibroblasts [18]. In this study it was shown that, regardless of the racial background of PCa cells, the degree of response (growth and/or proliferation) was significantly enhanced in experiments containing fibroblasts from AA patients.

Activated fibroblasts in the TME promote tumor progression by constant communication not only with cancer cells but also with other cellular components including an intimate regulation of immune-inflammatory cells [61]. CAF secrete a repertoire of growth factors and cytokines that contribute to cancer cell proliferation, invasiveness and angiogenesis [41]. The upregulation of genes involved in pathways and processes related to inflammatory response, immune response, and cytokine signaling in AA tumors compared to EA tumors indicate distinct immune profiles that could contribute to racial disparities [22]. The increased secretion of pro-inflammatory mediators from stromal cells induce the proliferation of PCa cells. CAFs isolated from prostate tumor stroma of AA patients secrete higher levels of pro-inflammatory cytokines and growth factors such as brain-derived neurotrophic factor (BDNF), VEGF, and fibroblast growth factor 7 (FGF7) compared to their EA counterparts [18]. PCa cell lines of AA origin have a more pronounced response to BDNF that translates to higher PCa cell proliferation and motility when compared to cell lines from EA. The influx of pro-inflammatory markers including stromal derived BDNF activate signaling pathways such as PI3K/AKT via tropomyosin receptor kinase B (TrkB) phosphorylation that may explain the aggressive nature of PCa in AA men [18]. The tumors from AA patients have a higher density of pro-tumorigenic immune cells and inflammatory cytokines compared to EA patients [63]. The prostate tumors of AA men showed a unique signature of pro-inflammatory cytokines, interferon-alpha (IFNα), IFNγ, tumor necrosis factor-alpha (TNFα), and Interleukin 4 and Interleukin 13 signaling that was associated with metastases and poor prognosis [63].

Inflammatory infiltrates represent another major component of the TME. TAM secrete a profile of growth factors and have been associated with growth and metastasis in several types of cancers including PCa [44,45]. AA patients with PCa have increased TAM compared to EA [18]. The infiltration of CD3+ T cells, CD68+, and CD163+ cells that are associated with macrophage polarization were significantly higher in prostate tissues from AA patients compared to EA [18]. Preliminary studies reported the upregulation of inhibitory proteins in CD4+T and CD8+ T cells, limiting T cell response in AA tumors and reducing immune cell function against tumor-associated antigens [64]. The presence of these infiltrating T cell lymphocyte subtypes induce an immune suppressive microenvironment, a known feature during carcinogenesis that blocks host anti-tumor response promoting tumor progression in Pca. A recent study showed that PCa tumors from an AA cohort had elevated numbers of infiltrating lymphocytes. These infiltrated lymphocytes are associated with higher proportion of plasma cells, NK cell activity and IFN γ signaling [65]. Patients with high plasma content showed improved survival following surgery [65].

Increased VEGF secretion by fibroblasts may impact tumor angiogenesis [18]. It has been shown recently that the TME of AA PCa exhibited higher microvascular density compared to EA patients TME [18]. These differences in TME could possibly affect tumor growth, maintenance, metastasis and response to treatment.

Genomic instability contributes to tumorigenesis and both germline and somatic mutations are amenable to novel therapeutic approaches. Somatic mutations of DNA repair and response mechanisms in PCa patients differ between AA and EA populations [66]. Although mutations of major DNA pathways were present in both AA and EA PCa tumors, AA men had a larger number of somatic mutations [66]. The consequences of these somatic alterations in cancer cells (or premalignant epithelial cells) on neighboring stromal partners including fibroblasts are currently not known. Whether this higher number (or specific pattern) of somatic changes observed in AA men is associated with an early onset of a tumor supportive TME remains to be determined. Unlike somatic mutations, germline alterations are present not only in the epithelium, but are in all cells in the body. These variants can potentially alter the function of a critical gene for a particular cell. For example, AA patients showed a distinct germline dinucleotide polymorphism, rs368234815 (TT or δG alleles) within the IFNL4 gene. This allele is associated with IFN-related damage resistance signature and predicts the overall survival rate of patients. rs368234815-δG in AA patients is associated with reduced survival [67]. In another study, it has been shown that the AA population has a high incidence of the nonsense SNP K1019X (A to T) on the EphB2 gene compared to EA populations [68]. K1019X is also found to be associated with increased risk of PCa in AA patients [68]. EphB2 is a tumor suppressive gene and in vitro studies showed significant reduction of proliferation and metastasis in the DU145 PCa cell line [69]. While the function of the K1019X SNP on stromal cell function is unknown the ephrin signaling system is active between the stromal and epithelial tissues and as such may play a role in mediating TME signaling to the tumor epithelium.

The expression of asporin (ASPN), an extracellular secreted protein with oncogenic potential [70] is elevated in the tumor stroma of PCa patients and, based on this observation, it has been proposed to be used as a CAF marker [71]. Moreover, polymorphisms in the N-terminus of ASPN were shown to be associated with PCa metastases, suggesting a role of ASPN in the TME in disease progression. Stromal expression of ASPN was associated with several clinical outcomes including higher Gleason Score, biochemical recurrence, and metastatic recurrence. The modulation of the germline ASPN D14 in the WPMY1 prostate fibroblast cell line was associated with increased metastasis of PCa cell line PC3 in vivo, whereas germline ASPN D 13 had a protective effect. The assessment of 1600 patients with localized PCa revealed that differences at the ASPN D locus were significantly and differentially linked to poorer oncologic outcomes [72]. Interestingly, AA patients that carry the ASPN D13 germline mutation were at a reduced risk of disease progression [72]. These studies give a glimpse into the potential role of genetic alterations in the stroma to disease outcome.

To date, there are only a limited number of functional studies focusing on the role of TME in PCa racial disparities. However, the evidence presented suggests that the TME in AA shelters a distinct tumor biology that not only promotes early onset or aggressive growth of PCa tumors but also inhibits anti-tumorigenic properties. Because the TME is considered genetically stable, it makes a more viable target for use in the prognosis or treatment of cancer. Future studies focusing on the role of particular cells or genes in the TME of PCa and how they contribute to tumorigenesis will aid in the development of personalized approaches in the management of PCa in racial disparities.

#### 3.1.2. Breast

Breast cancer is the second most common malignancy in US women, with more aggressive clinical presentation at the time of diagnosis and worse prognosis in AA patients compared to their EA counterparts [73]. Although the incidence of breast cancer is similar in these racial groups, the mortality rate is higher among AA populations compared to EA [74]. A different scenario is seen in the US with another minority group, Hispanic/Latina women, who have a lower incidence of breast cancer compared to the other two races. The decreased risk of developing breast cancer among this minority group could be due to unique reproductive characteristics in these women, with high parity, early age at first pregnancy, and high breastfeeding rates [75].

There are four subtypes of breast cancer based on the expression of estrogen and progesterone receptors (ER and PR) and epidermal growth factor receptor 2 (HER2). About 70% of breast cancer tumors are Luminal A (ER+/PR±/HER2-) with triple negative, commonly referred as TNBC (ER-/PR-/HER2-), and Luminal B (ER+/PR±/HER2±) accounting for 10% each. HER2-enriched (ER-/PR-/HER2+) make up around 4% of cases and unknown cases account for about 8% of tumors. Regardless of the race and age, Luminal A breast cancer is slow growing and is the most common subtype of breast cancer. However, AA women are at a higher risk of developing the more aggressive TNBC, whereas EA women are more susceptible to luminal subtype A [76]. The risk of breast cancer also varies with age. Regardless of the subtype, middle aged women (50–59 years) of either race are at higher risk of the disease [76]. Differences in tumor characteristics contribute to increased risk of breast cancer in middle-aged AA women compared to their EA counterparts [77].

Similar to PCa, the TME of breast cancer displays a molecular signature that significantly differs between AA and EA patients [20,54,58,78]. For example, the expression of Acyl-CoA oxidase 2 (ACOX2) and Mucin 1 (MUC1) genes were found to correlate with good prognosis. These genes were enriched in breast tumors from EA women compared to AA women [79]. In a recent gene expression study, Martin et al. extracted stromal areas of primary breast tumors using laser capture microdissection (LCM) and found higher expression of PSPHL and CRYBB2 genes in AA patients compared to EA [54]. According to one study, PSPHL expression differences in tumor epithelium between races are due to polymorphism on chromosome 7p11 with no apparent link between PSPHL levels and tumorigenesis in breast cancer [80]. However, in a different study, higher PSPHL expression was linked to metastases and a poor prognosis [79]. These discrepancies suggest that more functional studies are needed to elucidate the role of PSPHL in breast cancer. Both CRYBB2 and its pseudogene CRYBB2P1 are expressed in higher amounts in AA vs. EA breast tumors [57]. The induced overexpression of these genes in vitro and in vivo promoted tumorigenesis in human breast cancer cells [57]. Moreover, the increased expression of CRYBB2 in TNBC cell lines enhanced cell proliferation, tumor growth, interleukin 6 (IL6) production and expression of a panel of genes associated with EMT and metastasis [57].

Interferons protect against diseases by activating immune responses, they can also modulate cancer cell proliferation and metastatic spread. Analogous to racial differences in PCa, breast tumors had distinct interferon signatures in AA patients [19,54]. Several interferon-related genes are involved in the activation of signaling pathways in the TME of breast cancer, promoting chemotaxis, angiogenesis, and metastasis [54,81]. In addition to interferon, the TME of AA women with breast cancer has a higher abundance of the pro-inflammatory markers IL6 and resistin compared to EA women which could contribute to racial disparities in clinical outcomes [19]. Resistin is involved in tumor cell progression, invasion, and metastasis and therefore contributes to aggressiveness of the disease [19]. Deshmukh et al. showed that in cultured breast cancer cells, resistin induces IL6 production [19]. Resistin also enhanced IL6-driven STAT3 phosphorylation promoting growth and invasion of breast cancer cells [19].

Dense tumor infiltrating lymphocytes (TILs) in breast cancer indicate good prognosis and serve as an important biomarker for prediction of cancer treatment efficacy [82]. Breast cancer TILs include a large portion of T cells (CD8+ and CD4+ T cells), and smaller portions of B cells and NK cells [83]. CD8+ T cell density is higher in AA breast tumors compared to EA women and is associated with improved clinical outcomes in the AA population [84]. While increased CD8+ T cell density is linked to an overall increase in breast cancer patient survival rates, CD8+ T cell exhaustion contributes to a poor prognosis due to decreased effector cytokine production. Compared to their counterparts, AA patients showed poor response to treatment due to increased exhausted CD8+ T cells [85]. Similar to PCa, TAMs have been shown to contribute to breast cancer progression. Compared to EA and Hispanic/Latina American women, breast cancer in AA patients exhibited more TAMs [54,86] with increased M2 macrophage infiltration associated with poor prognosis in both AA and EA women [86]. The density of highly proliferative immunosuppressive macrophages (M2) is higher in the TME of AA women with breast cancer, whereas pro-inflammatory (M1) macrophage density is increased in EA women [86]. The expression of genes associated with angiogenesis is enriched in the tumor stroma of breast cancer patients [54]. Microvascular density, a measure of angiogenesis, is higher in the breast TME of AA patients compared to EA patients [54]. The increased macrophage infiltration and microvascular density in breast cancer are typically associated with tumor progression, metastasis and poor prognosis [87,88].

Overall, there is a large body of research showing race as a modulator of a distinct breast TME. Studies of TME associated with racial disparities suggest potentially useful signatures to aid in patient care by identification of novel therapeutic targets. In this review, we discussed the specific differences of breast TME components between two races (AA vs. EA) and the mechanisms by which TME components contribute to tumor progression and poor prognosis.

#### 3.1.3. Ovarian and Uterine Cancers

Racial disparities in various gynecological cancers result in higher mortality among AA women compared to other races [89]. Although socio-economic factors were shown to contribute to these differences, they do not fully account for these disparities. For example, in a study with equal access to healthcare providers, after adjustment for treatment and prognostic characteristics, AA women showed poor survival rates compared to other races [90]. This suggests the potential role of other factors, perhaps those associated with tumor biology, as contributing to the poor prognosis in AA women. Endometrial cancer is the second most prevalent cancer in women in the US and AA women are at high risk of advanced stage and high-grade disease at initial diagnosis [91,92]. Increasing evidence shows genetic and molecular alterations contributing to racial differences in endometrial cancer [93]. Transcriptomic data suggested significant differences in expression of PSPHL, SERPINA4, ITGA3, BET1L, and FAM228B between EA and AA women [56,94]. A comprehensive study looking at racial disparities in molecular subtypes of endometrial cancer revealed prevalence of aggressive molecular subtypes in AA women [95]. Utilizing the genomic data, three molecular subtypes of endometrial cancer were characterized by TCGA [96]. They are based on assessment of microsatellite instability status (MSI), copy number variant (CNV) calls, and somatic copy number alterations (SCNA). Among these subtypes, aggressive CNV high, SCNA 4 and mitotic subtypes are aggressive forms of endometric cancer. All three subtypes were prevalent in AA patients and associated with poor survival rate in AA compared to EA women [95]. Both AA and EA patients with mitotic subtype showed poor survival rate. However, AA women had worse prognosis compared to EA women. Within each molecular subtype, the cell cycle signaling pathways were significantly different between the two races [95]. The differential cell signaling pathways particularly in mitotic signaling between the two races indicate the race specific genomic characterization of the disease. The role of TME on health disparities in gynecological malignancies is still incompletely understood but current studies provide evidence of significance and demonstrate the need to explore TME contribution to these differences.

### 3.2. Digestive System

Colorectal is the third most common cancer in the US with the highest incidence and mortality rate among AA populations compared to other races [97]. The expression of genes that mediate inflammatory and immune response pathways in TME of colorectal cancer significantly differs between AA and EA populations [55]. Similar to breast and PCa, AA men with colorectal cancer had higher expression of CRYBB2 and PSPHL [55], and lower expression of immuno-inhibitory genes [21]. In addition to gene expression profile changes, genetic polymorphism differences exist between AA and EA populations [98]. Specifically, in colorectal cancer, Datta et al. reported an exonic SNP, rs34149860, in chromosome 3 associated with racial disparities [99]. rs34149860 is commonly found in AA patients with colorectal cancer and decreased levels of cohesin subunit 1 (SA-1) expression [99]. The presence of rs34149860 impacted the binding of miR-29b1 inhibitor overall affecting SA-1 expression in colon cancer. Differential expression of miR-29b has been associated with various disorders including fibrotic diseases, cancers, and neurodegenerative diseases via regulation of ECM proteins and pathways targeting collagens, fibrillins, and elastin [100].The TME of colon tumors have shown increased antitumor activity in EA compared to AAs [101] with lower density of CD8+T cells, macrophages and B cells [21]. CD8+T cells exhaustion is enhanced whereas Granzyme B expression, a measure of cytotoxicity activity in cells, is expressed at lower levels in the colon TME of AA patients compared to their EA counterparts [101,102]. In addition, cytokines such as Interleukin 10 and Interleukin 12 are depleted, and the expression of myeloid cells and mast cells increased resulting in an enhanced immunosuppressive environment in the TME of AA patients with colon cancer [101]. Racial disparities in the immune TME of patients with colon cancer could possibly explain survival outcomes differences between races.

### 3.3. Urinary System

The most common type of kidney cancer is renal cell carcinoma with clear cell renal cell carcinoma (ccRCC) being the most common histological form. The incidence of ccRCC is higher among AA compared to EA [2]. The TME of renal cell carcinoma differs between AA and EA patients with differential TIL composition and abundance. The abundance of follicular helper and regulatory T cells significantly increased with disease stage among EA patients. Regulatory T cells are involved in modulating the response to checkpoint inhibitor immunotherapy and differences in TME between AA and EA might result in differential response to immunotherapy treatment in renal cell carcinoma patients with advanced stage disease [103].

### 3.4. Respiratory System

Lung cancer is the leading cause of cancer death in the US and AA populations have high incidence and mortality rates compared to all other races [1,2]. Non-small cell lung cancer (NSCLC) is the most common form of lung cancer comprising >80% of tumors. NSCLC tumors in AA patients were shown to have higher fractions of follicular helper T cells, gamma delta T cells, M1 macrophages, and M2 macrophages compared to lung tumors in EA patients [104]. Chromosomal aberrations and mutations lead to genetic heterogeneity and there is a notion that the TME contributes to genetic instability in cancer cells and further promotes tumorigenesis. PTPRT and JAK2 mutations (for example) are seen at higher frequency in AA lung tumors compared to EA patients [105]. However, the role, if any of TME in this acquisition of mutations is not known. Future research will determine whether racial differences of the tumor stroma are associated with tumor mutational burden.

Overall, these studies showed that AA patients host a unique immunosuppressive environment that may promote, or allow, the emergence of aggressive forms of various types of cancers. A better understanding of the AA immune signature in cancer tissues may help the development of personalized immunotherapy treatment strategies in this population. While TME has been shown to play a key role in cancer growth and progression in many studies, it has received less attention when it comes to the arena of racial disparities. Although at its infancy, the scientific literature of TME in racial disparities in various cancers is starting to point at the stroma as a focus of future studies to elucidate the biology aiming at personalized therapeutics to tackle racial disparities in cancer. In recent years, a large arsenal of novel drugs has been developed but has not been evaluated for their use in cancer disparities. Although only a few targets and mechanisms were presented in this review, in the following section we will discuss some drugs already approved for their use in the clinic or in development that could potentially be used in preclinical or clinical trials.

### 3.5. Potential Therapeutic TME Targets in Racial Disparities

Current therapeutic approaches against malignant tumors are based on the clinico-pathological characteristics of cancer cells and, depending on stage and grade, typically include a variety of options from surgery, chemotherapy or radiation therapy alone or in combination as the first treatment strategies [106]. Newer immunotherapy approaches are now also becoming available as early treatment options in some cases [107,108]. The discovery of key mutations in proteins such as epidermal growth factor receptor (EGFR), p53, and c-Myc and their association with carcinogenesis led to an explosion of drug development targeting these molecules to combat cancer [109]. Unfortunately, these strategies resulted in cancer cells acquiring drug-resistance leading to tumor relapse or poor quality of patient life [109]. Our incomplete understanding of the mechanisms for therapeutic resistance in combination with many recent studies highlighting the importance of the TME for successful delivery of drugs to cancer cells suggest that the tumor stroma might provide some answers to this problem.

Despite substantial differences in the incidence and progression of different tumors between races, there are no tailored therapeutic interventions to counter these cancer health disparities. The TME has an effect not only on tumor growth and progression but also has a significant impact on drug resistance and clinical outcomes [110,111]. Targeting the TME is now considered an integral part of active anticancer strategies, as shown by several successful drugs currently in clinical use. For example, small molecule inhibitors have high specificity and penetrating capacity to the target within tumors [112]. A large number of small molecule inhibitors or activators targeting CAFs, immune and inflammatory signaling pathways in TME have shown promising results with beneficial outcomes in cancer treatment [113]. Despite the marked differences in TME, there have been no clinical studies comparing the efficacy of these small molecule inhibitors between AA and EA patients. Here, we will discuss some FDA approved drugs with potential benefits in the treatment of cancers associated with racial disparities that target aspects of the tumor stroma.

The FDA has already approved a few TME-targeting therapeutics to be used in conjunction with other treatment strategies. For example, Bevacizumab (Avastin), an antibody that targets VEGF, was the first anti-angiogenic drug approved by FDA in combination with chemotherapy to treat several types of cancers including metastatic colorectal cancer, NSCLC, Glioblastoma, mRCC, and cervical cancer. In a clinical trial to study the efficacy of Bevacizumab, in combination with other chemotherapy drugs (doxorubicin hydrochloride, cyclophosphamide, and paclitaxel) used in breast cancer included mixed ethnicity with 2473 EA and 386 AA women (NCT00433511). This is an active and ongoing phase III clinical trial with a secondary outcome to study the association between invasive disease-free survival (IDFS) rate and race. Other VEGF receptor targeted drugs followed Bevacizumab and include Pazopanib, Ramucirumab, and Aflibercept for the treatment of different types of cancers. Afinitor (Everolimus) is another neovascularization targeted drug that binds to immunophilin FK Binding Protein-12 (FKBP-12) and inhibits mammalian Target of Rapamycin (mTOR) [114]. The inhibition of mTOR reduces endothelial cell proliferation by blocking mTOR/VEGF pathway [114].

Several clinical trials are in progress to use these and other drugs targeting angiogenesis in various cancer treatments in combination with chemotherapy and immunotherapy (Table 1). Pexidartinib (Turalio), which inhibits the colony stimulating factor 1 receptor (CSF1R), is used for the treatment of symptomatic tenosynovial giant cell tumor (TGCT). CSF1R inhibition reduces the number of M2 polarized macrophages in the TME and reprograms the remaining TAMs into an M1 phenotype to promote antigen presentation and T cell activation [115]. Several pre-clinical studies have shown fibroblast activation protein alpha (FAP) modulation as a key approach to inhibit fibroblast differentiation into CAFs. RO6874281 is a recombinant fusion protein that consists of anti-FAP linked to engineered variant of IL-2v and is a potential immune stimulator [116]. This fusion protein has shown promising outcomes in phase I and phase II clinical trials of several cancers including breast, head and neck, renal and pancreatic cancers (NCT02627274, NCT03875079, NCT03063762, NCT03193190). Currently, there are several ongoing active clinical trials targeting these components of TME either as alternatives or adjuncts to chemotherapy/radiation therapy (Table 1 and Table 2).

Other potential TME targets that are in clinical trials include TGFβ pathway inhibitors, blocking IL-1βR, IL6R, CXCR4 downstream signaling pathways, tyrosine kinase inhibitors, and checkpoint inhibitors like programmed cell death ligand 1 (PDL1) (Table 1 and Table 2). As we discussed earlier, some of these molecules/pathways have differential expression profiles in the AA and EA TME of various cancers, therefore they are considered potentially attractive therapeutic targets for personalized medicine. For example, in a retrospective study, co-expression of programmed cell death ligand 1 (PD-L1) and Indoleamine 2,3 dioxygenase (IDO) was linked to higher levels of immune infiltration in AA patients with high-grade serous ovarian carcinoma (HGSOC) [117]. This study indicates that treating AA women with ovarian cancer with a combination of PD-L1 and IDO inhibitors could be beneficial [117].

Patient stratification with the inclusion of race as a confounder should be encouraged in clinical trials to study the benefits for racial disparities in the diagnosis and treatment of several types of cancer. AAs are underrepresented in FDA clinical trials for cancer therapies accounting for only ~7.5% of total participants for all cancers combined vs. a 13% representation in the general U.S. population [118]. Several cancers such as prostate, breast, gynecological, colorectal and lung have high incidence and/ or mortality in AA population, however the selection of the drug and/or treatment approaches are usually based on studies that include a disproportionate large number of EA patients and might differ for a similar AA population.

## 4. Conclusions

In this review, we highlighted some underlying molecular differences in the TME as potential key drivers of health disparities in cancer. There are strong indicators of racial disparities in the TME of various cancers that should be considered while choosing treatment strategies. The AA population shows a unique signature of cancer vulnerability in their TME compared to its counterpart EA population due to its highly immunosuppressive properties. These differences may contribute to the risk of cancer development, aggressive form of disease, and response to therapy. Due to significant enrichment of genes and pathways leading to aberrant immunosuppression in the TME of AA, modulating the immune system by immunotherapy treatment strategies in these patients may render more benefits than the current treatment approaches. Although racial differences in the components and mechanisms within the TME are evident, to date there have not been studies validating the utility of these molecules for diagnostic purposes or therapeutic interventions. Acknowledging the importance of TME in racial disparities is paramount to future basic and clinical research studies focused on personalized cancer diagnosis and treatment.

## Figures and Tables

**Table 1:** Therapeutic agents targeting tumor microenvironment components/pathways in interventional phase 3 and 4 clinical trials currently active and recruiting or not yet recruiting. Data acquired from the USA National library of medicine (http://clinicaltrials.gov, accessed on 16 March 2021).

Target	Drug Name	Cancer Type	Clinical Trial #	AA ^1^ vs. EA ^2^ Percentage (n) of Study Participants
VEGF ^3^	Bevacizumab	Non-small cell lung	NCT01107626	11.9% (180) vs. 84% (1273)
			NCT00946712	8.8% (116) vs. 86.3% (1133)
			NCT00324805	8.7% (131) vs. 86.7% (1302)
		Neuroendocrine	NCT00569127	9.5% (38) vs. 83.6% (336)
		Colon cancer	NCT00217737	No data available
			NCT00109070	No data available
			NCT02997228	No data available
		Urinary Tract	NCT00942331	3.6% (18) vs. 91.7% (464)
		Ovarian, fallopian tube, Peritoneal	NCT01167712	No data available
			NCT01081262	No data available
			NCT00565851	4.2% (44) vs. 64.8% (682)
			NCT00951496	3.3% (51) vs. 91.5% (1427)
		Breast	NCT00109239	No data available
			NCT00028990	No data available
			NCT01663727	No data available
			NCT00433511	7.7% (386) vs. 49.5% (2473)
			NCT00601900	No data available
			NCT00785291	14.1% (113) vs. 80.1% (640)
	Aflibercept	Ovarian	NCT00327444	1.8% (1) vs. 74.5% (41)
	Zometa	Breast	NCT00524849	No data available
	ZACTIMA	Non-small cell lung	NCT00312377	No data available
	Regorafenib	Colorectal	NCT03564938	No data available
	Fruquintinib	Colorectal and Colon	NCT04322539	No data available
	Lenvatinib	Endometrial	NCT03517449	No data available
	Cediranib	Ovarian	NCT03278717	No data available
IL-1β ^4^	Canakinumab	Non-small cell lung	NCT03626545	No data available
			NCT03631199	No data available
			NCT03447769	No data available
anti- PDL1 ^5^	Atezolizumab	Triple negative Breast	NCT03498716	No data available
			NCT03125902	4.8% (31) vs. 57.5% (374)
			NCT02425891	No data available
			NCT03498716	No data available
			NCT03371017	No data available
			NCT03197935	No data available
			NCT04177108	No data available
		HER2 positive breast	NCT04740918	No data available
			NCT03726879	No data available
			NCT03199885	No data available
		metastatic castration-resistant prostate	NCT03016312	No data available
			NCT04446117	No data available
		Non-small cell lung	NCT04513925	No data available
			NCT02657434	1% (6) vs. 68.5% (396)
			NCT02409342	0.7% (4) vs. 83.7% (479)
			NCT04471428	No data available
			NCT03456063	No data available
			NCT03178552	No data available
		Small cell lung	NCT04256421	No data available
			NCT02763579	0.7% (3) vs. 79.9% (322)
			NCT03811002	No data available
		Lung	NCT02486718	No data available
		Renal cell carcinoma	NCT04338269	No data available
			NCT02420821	0.5% (5) vs. 72.1% (660)
		Ovarian, fallopian tube, Peritoneal	NCT03038100	No data available
		Bladder	NCT03775265	No data available
		Colon	NCT02912559	No data available
		Urothelial Carcinoma	NCT02807636	No data available
Immunotherapy (Macrophages)	Sipuleucel-T	Prostate Adenocarcinoma	NCT03686683	No data available

^1^ AA-African Americans, ^2^ EA-European Americans. ^3^ VEGF-Vascular endothelial growth factor, ^4^ IL1β-interleukin 1 beta, ^5^ PDL1- Programmed cell death ligand 1.

**Table 2:** Therapeutic agents targeting few tumor microenvironment components/pathways in interventional phase 1 and 2 clinical trials currently active and recruiting or not yet recruiting. Data acquired from the U.S. National library of medicine (http://clinicaltrials.gov, accessed on 16 March 2020).

Drug Name	Target	Status	Cancer Type	Clinical Trial #
Tocilizumab	anti–IL6R	Phase 2	Non-small cell lung cancer	NCT04691817
				NCT03337698
			Prostate cancer	NCT03821246
			Head and Neck Cancer	NCT03708224
			Liver cancer	NCT04524871
			Triple negative Breast cancer	NCT03424005
			Morpheus-Pancreatic Cancer	NCT03193190
			Bladder Cancer	NCT03869190
			Melanoma	NCT03999749
		Phase 1	HER2 positive Breast cancer	NCT03135171
			Colorectal cancer	NCT03866239
Plerixafor	CXCR4	Phase 2	Metastatic Pancreatic Cancer	NCT04177810
			Multiple Myeloma	NCT04552743
			Malignant Gliomas	NCT00669669
			Glioblastoma	NCT03746080
Vigil	TGFβ1 and TGFβ2 suppressor	Phase 2	Advanced Gynecological Cancers	NCT03073525
AVID200	TGFβ pathway inhibitor		Myelofibrosis	NCT03895112
RO6874281	Fibroblast Activation Protein-α (FAP)	Phase 1	Metastatic Melanoma	NCT03875079
			Breast Cancer, Head and Neck cancer	NCT02627274
			Metastatic Renal Cell Carcinoma	NCT03063762
			Pancreatic Adenocarcinoma	NCT03193190
Emactuzumab	CSF1R	Phase 2	Ovarian, fallopian tube, Peritoneal	NCT02923739

IL6-interleukin 6, CXCR4-C-X-C Motif Chemokine Receptor 4. TGFβ1-Transforming Growth Factor Beta 1, TGFβ2-Transforming Growth Factor Beta 2. TGFβ-Transforming Growth Factor Beta, CSF1R-Colony Stimulating Factor 1 Receptor.
